# Biopolitics and the Ethical Dilemmas of Biotechnology: Lessons From the Pandemic

**DOI:** 10.1111/1751-7915.70114

**Published:** 2025-10-28

**Authors:** Ioannis Economidis

**Affiliations:** ^1^ European Commission, Directorate‐General Research and Innovation (Ret.) Brussels Belgium

**Keywords:** bioeconomy, biopolitics, biotechnology, climate change, COVID, human rights

## Abstract

The COVID‐19 pandemic has underscored the urgent need for sustainability‐driven biotechnology that strikes a balance between economic development, environmental stewardship and social impact, all while upholding fundamental human rights.

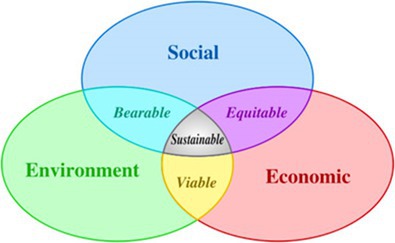

The COVID‐19 pandemic was not just a public health crisis; it was also a moment of profound societal transformation. It forced governments, scientists and the general public to confront difficult questions about the relationship between science and governance, ethics and technology, security and personal freedom. Although biotechnology has long promised solutions to medical, agricultural and environmental challenges, the pandemic made it clear that its application is never neutral—it is always embedded within political and social structures. A key concept that emerged during this period is *biopolitics*, a term describing the intersection of human biology and political governance. The outcomes of the pandemics were mixed: Although some policies successfully curbed the spread of disease, others led to social alienation, distrust in institutions and an erosion of democratic norms. The pandemic, in effect, was the largest global exercise in biopolitical regulation in history.

The experience of COVID‐19 has divided society into two historical phases—what we might call *Pre‐COVID* and *Post‐COVID* worlds. In the pre‐pandemic world, scientific progress was often seen as a force for democratisation and equality, a way to enhance human well‐being. The post‐pandemic reality, however, has shown that science and technology can also be wielded as tools of control, surveillance and exclusion. One of the most striking aspects of this divide is the shift in public perception regarding authority and expertise. Scientific knowledge was both highly valued and deeply contested. On one hand, rapid vaccine development demonstrated the power of biotechnology and global cooperation. On the other, widespread misinformation and public scepticism revealed deep fractures in trust between governments, scientists and citizens. Though rooted in political science and philosophy, the pandemic brought biopolitics into everyday life. Governments around the world had to make difficult decisions, balancing public health with economic stability, individual rights with collective safety. This transformation has significant implications for future governance. COVID‐19 demonstrated how states can exert control over populations in ways that were previously unimaginable—restricting movement, mandating medical interventions and implementing digital tracking systems. Although some of these measures were necessary for public health, they also set new precedents for governmental power over citizens' life. These developments raise fundamental questions: What are the limits of state intervention in personal health? How can scientific advancements be deployed without compromising civil liberties? The biopolitical divide of pre‐ and post‐COVID societies has thus become evident.

## 1 Biopolitics, Eugenics and the Ethics of Decision‐Making

The pandemic also exposed a troubling ethical dilemma: how societies determine who receives medical treatment in times of crisis. In some cases, hospitals faced resource shortages that forced them to prioritise certain patients over others. These decisions, while often made under extreme conditions, echoed historical forms of eugenics—where some lives were deemed more valuable than others. The distinction between *zoe* (bare biological life) and *bios* (life within a society) became crucial in this context. Michel Foucault's concept of *biopower*—the idea that governments regulate life itself—was evident in the way certain populations were marginalised. Giorgio Agamben expanded on this by describing how crises reduce individuals to mere biological entities, stripped of their political and social rights.

These concerns are not merely theoretical; they have real‐world consequences. The early days of the pandemic saw disparities in healthcare access, vaccine distribution and economic relief. Those in marginalised communities often bore the brunt of the crisis, reinforcing existing inequalities. If biotechnology is to fulfil its promise of improving human life, it must be applied with an awareness of these ethical challenges.

## 2 The Triangle of Science, Ethics and Economics

**FIGURE 1:**
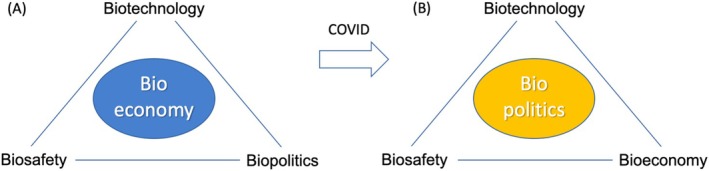
The concept of the bioeconomy emerged long before the pandemic as a proposal to replace the large‐scale chemical and manufacturing industries—which define our society but come at a significant environmental cost—with alternatives based on biological processes. This concept is built on major advances in biotechnology, coupled with care about the safety of the new procedures and the oversight of their political implications. However, in the wake of COVID‐19, it has become evident that biopolitics is not a peripheral aspect of the bioeconomy but rather its core, poised to shape key decisions and directions in the biotechnological field for years to come.

Scientific progress does not exist in a vacuum. It operates within a dynamic framework that can be visualised as a triangle (Figure 1A), with three interdependent forces: [i] *Economic value*—Science and technology drive economic growth, and in turn, require financial investment to advance. However, market forces can sometimes prioritise profit over societal well‐being. [ii] *Ethical, legal and social (ELS) values*. The governance of biotechnology must be guided by ethical principles, human rights protections and legal safeguards. Without these, scientific progress risks being exploited for harmful ends. [iii] *Environmental sustainability*. Biotechnology increasingly plays a role in addressing global challenges such as climate change, food security and water scarcity. Yet, without proper regulation, technological advances can also contribute to environmental degradation. Maintaining balance within this triangle is crucial. If economic forces dominate, ethical considerations may be sidelined. If regulations are too rigid, scientific innovation may be stifled.

If environmental concerns are ignored, the long‐term viability of technological progress is threatened. The COVID‐19 crisis demonstrated how fragile this equilibrium can be—and how essential it is to maintain. For this to happen, biopolitics is bound to have a central role to balance the equilibrium among different forces that shape development of contemporary Biotechnology (Figure 1B).

## 3 Biotechnology and Human Rights

Legal frameworks, particularly in the European Union, provide essential protections in biotechnology and medical ethics. The Charter of Fundamental Rights ensures the right to bodily integrity and informed consent in medical treatments, the prohibition of eugenics and genetic discrimination, the banning of financial exploitation of the human body and the protection of academic freedom and independent research. These principles serve as a safeguard against the misuse of scientific advancements. However, their enforcement is not always guaranteed. The pandemic revealed how easily ethical norms can be compromised in the face of emergency decision‐making. Moving forward, policymakers must ensure that crisis responses do not erode fundamental human rights.

## 4 Climate Change, Bioeconomy and the Next Global Challenges

The COVID‐19 pandemic is not the last crisis humanity will face. Climate change, food insecurity, water shortages and energy demands are already shaping the future. The European Commission has warned of a *Perfect Storm* by 2050—an intersection of global crises that will require coordinated international action. Biotechnology will play a crucial role in addressing these challenges. Innovations in bioenergy, precision agriculture and synthetic biology hold promise for sustainable solutions. However, these technologies must be developed within an ethical framework that prioritises equity and environmental responsibility. As Mahatma Gandhi famously stated, ‘… the Earth provides enough to satisfy every man's need, but not every man's greed…’. This wisdom is particularly relevant in discussions of biotechnology. Scientific advancements should serve humanity as a whole, not just the interests of a select few. Achieving this goal will require international cooperation, transparent governance and a commitment to ethical science.

## 5 The Role of Education, Culture and Public Engagement

Beyond policy and regulation, a key factor in shaping the future of biotechnology is public engagement. Scientific literacy must be strengthened to combat misinformation and foster informed decision‐making. Academic freedom must be protected to ensure that researchers can pursue knowledge without political or corporate interference. Cultural perspectives must also be considered, recognising that science does not exist in isolation from societal values and traditions. A balanced approach to science and governance requires not only technical expertise but also philosophical reflection. As Sophocles wrote in *Antigone*, “Wisdom is the foundation of happiness.” True progress is not measured solely by technological achievements, but by how well we integrate knowledge with ethical responsibility.

## 6 Conclusion: Toward a More Just and Sustainable Future

The pandemic has reshaped our understanding of biotechnology, ethics and governance. It has revealed both the strengths and weaknesses of existing systems, forcing us to confront difficult questions about the role of science in society. Moving forward, we must strive for a future where biotechnology serves humanity without reinforcing inequality or compromising fundamental rights. This requires maintaining balance within the triangle of economics, ethics and sustainability. It demands also vigilance against the misuse of biopolitical power. And above all, it calls for a renewed commitment to international cooperation and responsible innovation. The challenges of the 21st century are complex, but they are not insurmountable. With wisdom, foresight and ethical leadership, we can ensure that biotechnology remains a force for good—one that empowers, rather than controls, human life.

## Author Contributions

**Ioannis Economidis:** conceptualization; writing – original draft.

## Conflicts of Interest

The author declares no conflicts of interest.

## Data Availability

Data sharing not applicable to this article as no datasets were generated or analysed during the current study.

